# Time Frame and Justice Motive: Future Perspective Moderates the Adaptive Function of General Belief in a Just World

**DOI:** 10.1371/journal.pone.0080668

**Published:** 2013-11-28

**Authors:** Michael Shengtao Wu, Robbie M. Sutton, Xiaodan Yan, Chan Zhou, Yiwen Chen, Zhuohong Zhu, Buxin Han

**Affiliations:** 1 School of Journalism and Communication, Xiamen University, Xiamen, China; 2 Institute of Psychology, Chinese Academy of Sciences, Beijing, China; 3 School of Psychology, Kent University, Canterbury, United Kingdom; 4 Department of Psychiatry, New York University, New York, New York, United States of America; 5 School of Psychology, Beijing Normal University, Beijing, China; George Mason University/Krasnow Institute for Advanced Study, United States of America

## Abstract

**Background:**

The human ability to envision the future, that is, to take a future perspective (FP), plays a key role in the justice motive and its function in transcending disadvantages and misfortunes. The present research investigated whether individual (Study 1) and situational (Study 2) differences in FP moderated the association of general belief in a just world (GBJW) with psychological resilience.

**Methodology/Principal Findings:**

We investigated FP, GBJW, and resilience in sample of adolescents (n = 223) and disaster survivors (n = 218) in China. In Study 1, adolescents revealed stronger GBJW than PBJW, and GBJW uniquely predicted resilience in the daily lives of those with high FP (but not those with low FP). In Study 2, natural priming of FP (vs. no FP) facilitated the association of GBJW with resilience after disaster.

**Conclusions/Significance:**

Supporting predictions, participants endorsed GBJW more strongly than PBJW. Further, GBJW interacted with FP in both studies, such that there was an association between GBJW and resilience at high but not low levels of FP. The results corroborate recent findings suggesting that GBJW may be more psychologically adaptive than PBJW among some populations. They also confirm that focusing on the future is an important aspect of the adaptive function of just-world beliefs.

## Introduction

Humans are endowed with the ability to envision the future and to anticipate the hedonic consequences of future events [1], [2]. This future perspective (FP) underlies the fundamental organizing principles of motivation, cognition, and affect [3], [4]. One of these principles is faith in justice [5], [6], [7]. As justice motive theory asserts, individuals are motivated to believe that they live in a world in which people get what they deserve [8], [9]. Theoretically, this belief in a just world (BJW) promotes the expectation that life will be stable and orderly, and that people can confront an otherwise uncertain future with confidence. Recent years have seen a growth of interest in the justice motive and how it relates to the human ability to envision the future [5], [6], [7], [10], [11], [12], [13].

One of the key findings of recent research on BJW is that it is closely related to mental health. Individuals with strong BJW often show considerable levels of resilience when coping with life stressors [14], [15], [16], [17]. However, this finding depends on whom is imagined to be the recipient of justice. The belief that one will personally receive justice (PBJW) is psychometrically and functionally distinct from the belief that people generally receive justice (GBJW). Several studies, conducted largely with WEIRD (Western, educated, industrialized, rich and democratic [18]) samples, have shown that PBJW is endorsed more strongly and associated more strongly with mental health than GBJW [19], [20], [21], [22]. However, this is not necessarily the case among less privileged Western populations [7], [23]. Further, GBJW has been found to be stronger than PBJW among disadvantaged populations (e.g., ordinary people in the developing China, India, and Armenia), and to promote resilience among people confronting adversities such as natural disasters [24], [25], [26], cancer or AIDS [27], and chronic pain [23]. Such findings challenge the theoretical consensus that people believe that the world is more fair to themselves than generally, and that they benefit more from this belief [28], [29], [30], [31].

Most important, the original theoretical statements of justice motive theory link future perspective with the psychological importance of GBJW for people who are enduring chronic disadvantage or suffering. People whose lives are currently imbued with injustice may derive some comfort from the belief that other people’s lives are just, since the justice experienced by others may portend that they themselves will experience more deserved outcomes in the future. In keeping with this reasoning, research has shown that disadvantaged (vs. advantaged) people benefit more than adopting beliefs in control that imply that just outcomes prevail for most people [32], [33]. In short, for people enduring unjust lives, faith that “other people get what they deserve” may promote the faith that “I will get what I deserve,” and so help them to cope with the adverse circumstances of their lives in the present day.

Other lines of research also link future perspective to the strength and function of faith in the justice of the world to people generally. Some studies suggest that GBJW is associated with defensive responses to victims, such as derogation, more strongly when people are focused on their long-term goals for the future [5], [6], [11], [13]. Other studies show that system-justifying beliefs, which promote the expectation that people generally get what they deserve, contribute to effective self-regulation and pursuit of long-term goals [33], [34].

In the present research, therefore, we are concerned with the interplay between future perspective and just-world beliefs. If GBJW enables people who live in relatively disadvantageous circumstances (e.g., relative to WEIRD participants), to confront the environment confidently [8], [9], we would expect it to be associated with psychological resilience – especially when these people have adopted a future perspective. Two studies with Chinese participants test whether GBJW is endorsed more strongly than PBJW, and more strongly associated with psychological resilience. They also examine whether the relationship between GBJW and psychological resilience is stronger among participants with a stronger focus on their future (i.e., higher FP). In Study 1, we examine the moderating effects of dispositional FP among middle school students. In Study 2, we examine the moderating effects of a situational proxy of FP among survivors of an earthquake, in which survivors who have been placed in new housing are assumed (and shown) to be higher in FP than those who are waiting to be placed in new housing.

## Study 1

In our previous studies, GBJW was found to be stronger than PBJW in childhood and adulthood, and to function as a psychological resource for coping with adversity [24], [25], [26], [27]. However, these studies did not examine the role of FP in the effect. In the present study, we recruited Chinese adolescents in order to examine the relative strength and function of GBJW, and to conduct the first empirical investigation of the role of FP. Researchers have suggested that just-world belief are of particular psychological importance in adolescence, since envisioning the future, pursuing long-term projects, and avoiding long-term pitfalls is especially important in adolescence [7], [35]. This suggests that just-world beliefs may be especially important among adolescents who are dispositionally high in FP. As found in previous studies with Chinese adolescents, we hypothesized that adolescents would have a greater GBJW than PBJW [26]. More importantly, we expected that FP would moderate the relationship of GBJW and resilience; in adolescents with high FP, GBJW would predict resilience, but in adolescents with low FP, GBJW would not.

### Method

#### Participants

Two hundred and twenty-eight adolescents in a middle school in Beijing were asked to complete the questionnaires, and 223 of them responded as instructed (12 to 15 years old, M = 13.27; 116 girls). The other five were identified as oversea students who were not able to fully understand the instructions and questions in Chinese, thus were not included in the further analysis. Written informed consent from the participants and their guardians was obtained, and this protocol was approved by the Institutional Review Board of Institute of Psychology, Chinese Academy of Sciences (No. 10007).

#### Materials and Procedure

A 13-item BJW scale was employed to assess both GBJW (6 items, e.g., “I believe that, by and large, people get what they deserve”, *α*  = .85) and PBJW (7 items, e.g., “I believe that I usually get what I deserve”, α = .87) [26], [30](1  =  strongly disagree, 6  =  strongly agree).

A 10-item resilience scale (α  = .90) was used to measure resilience, which is positive adaptation to daily life stress (e.g., “I tend to bounce back after illness or hardship”) [36], [37] (0  =  not true at all, 4  =  true nearly all the time).

FP was measured by three items (α  = .81): “I consider how a situation will develop in the long run to formulate an appropriate strategic approach”, “I will adjust my approaches according to future changes” and “I will take a long-term perspective to evaluate my gains and losses”, 0  =  never, 4  =  always). These items were from a Chinese long-term orientation scale concerning the future and past perspective [38], and readability in simplified Chinese was taken into consideration in selecting the items.

The questionnaire was distributed and completed in the classroom. A small gift (four issues of a newspaper about middle-school students’ health) was offered as reward. The data collection process was conducted under the supervision of the adolescents’ teachers.

### Results

As presented in Table 1, both GBJW and PBJW were positively correlated with FP and psychological resilience. Furthermore, a paired-sample *t*-test showed that GBJW (*M* = 4.28±1.12) was stronger than PBJW (*M* = 4.16±1.04), *t*(221)  = 2.11, *p*  = .036.

**Table 1:** Correlation of GBJW and PBJW with future perspective and resilience in Study 1 and Study 2.

	Ordinary Adolescents			Disaster Survivors	
	GBJW	PBJW		GBJW	PBJW
PBJW	0.68**			0.55**	
Gender	0.11	–0.00		–0.06	0.06
Age	0.02	0.05		0.13*	0.21**
FP	0.24**	0.25**		0.19**	0.11
Resilience	0.33**	0.31**		0.16*	0.10

=  General Belief in a Just World, PBJW  =  Personal Belief in a Just World, FP  =  Future Perspective. *p < 0.05, **p < 0.01. Note. GBJW

Hierarchical regression analyses were used to test the moderation effect of FP on GBJW (vs. PBJW) and resilience, in which the mean-centered interaction term (FP × GBJW or FP x PBJW) was entered into the regression model after demographic variables, while FP and GBJW (or PBJW) were controlled. Results of the first hierarchical model concerned with GBJW showed that as predicted, resilience was significantly predicted by FP × GBJW, *β* = 0.19, *t* = 3.38, *p*  = .001, as well as GBJW, *β* = 0.20, *t* = 3.47, *p*  = .001, and FP, *β* = 0.47, *t* = 8.27, *p*  = .000. In contrast, the second hierarchical showed that while resilience was predicted by both PBJW, *β* = 0.19, *t* = 3.21, *p*  = .002, and FP, *β* = 0.46, *t* = 7.72, *p*  = .000, there was no evidence of moderation since there was no significant effect of FP × PBJW, *β* = 0.04, *t* = 0.62, *p*  = .538.

**Figure 1:**
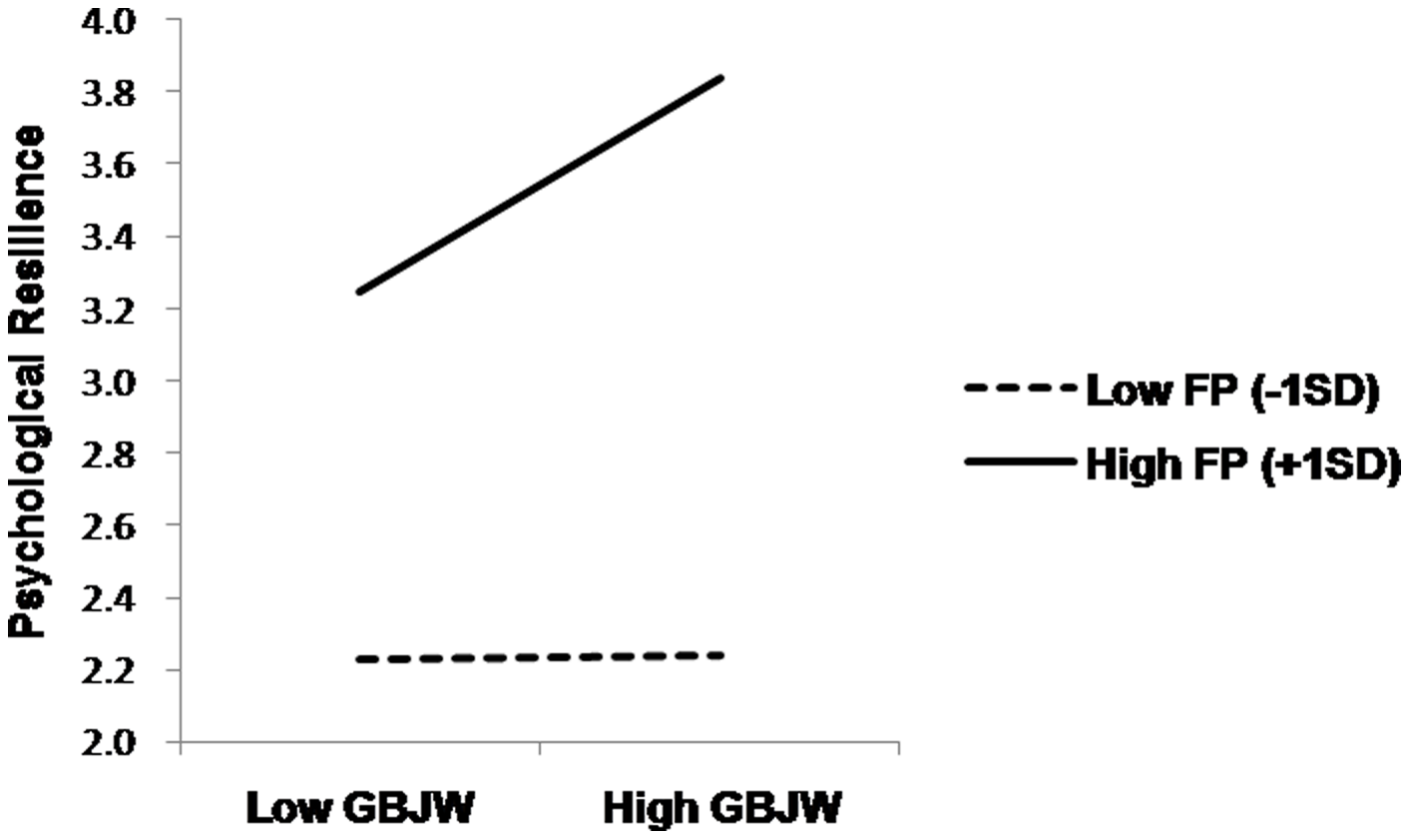
Relationship of adolescents’ GBJW and psychological resilience, depending on the variance of dispositional FP (Study 1). GBJW  =  general belief in a just world, FP  =  Future Perspective.

As shown in Figure 1, we demonstrated the interaction effect of FP and GBJW on psychological resilience, by comparing the simple slopes [39] relating GBJW to psychological resilience among adolescents with high FP (+1 SD) and low FP (–1 SD). As expected, GBJW, *β* = 0.66, *t* = 3.09, *p*  = .004, but not PBJW, *β* = –0.11, *r* = –0.52, *p*  = .605, predicted resilience among those with high FP. Among those low in FP, GBJW *β* = –0.45, *t* = –1.90, *p*  = .067, and PBJW, *β* = 0.43, *t* =  2.00, *p*  = .054, marginally predicted resilience.

### Discussion

Several interesting findings emerged from this study. First, we replicated our earlier findings suggesting that GBJW is stronger than PBJW among Chinese adolescents [26]. Second, we showed that GBJW is positively associated with psychological adjustment – specifically, with resilience – among these adolescents. Third, and most novel, we showed that the positive relationship between GBJW and resilience was moderated by participants’ temporal perspective. It was robust among adolescents who had adopted a high FP, and marginal among adolescents with low FP. In the next study, we examine whether this moderation effect extends beyond adolescence into adulthood, and also whether it holds for participants who are confronting especially challenging or unjust life circumstances – specifically, those whose homes were destroyed by a natural disaster.

## Study 2

In Study 2, we focused on the interplay between FP and GBJW among those experiencing harsh realities, in which resilience should be crucial for survival and adjustment. Natural disasters are vivid and tragic examples of such realities, exposing people to danger, disorder, and uncertainty. Previous research suggests that GBJW may boost resilience for people facing a disaster by offering a hopeful future [24], [25], [26]. In the present study, we hypothesized that GBJW would have this function if there was a tangible asset in the future. For example, as the overriding concern of survivors after the 2008 Wenchuan earthquake, some of the survivors moved into new permanent apartments. In contrast, we propose that the function of GBJW would break down if hope was not tangibly reinforced long after the disaster, for example, for those survivors who remained long-term in temporary shelters.

### Method

#### Participants

Two hundred and thirty six survivors were recruited to take part in the survey 26 months after 2008 Wenchuan Earthquake in China, and 218 of them (*M* = 42.17±16.98, from 18 to 78 years) were identified to be local residents who experienced the earthquake and accepted the invitation to participate. Written informed consent from the participants was obtained, and this protocol was approved by the Institutional Review Board of Institute of Psychology, Chinese Academy of Sciences (No. 10007).

All participants were forced to move out of their houses by the earthquake. At the time the survey was taken, 112 had moved to new apartments (*M* = 42.17±17.90 years; 51 women), and 106 still lived in the temporary shelters of the disaster (*M* = 42.18±16.03 years; 51 women). Two groups were comparable in age, *t*(216)  = 0.00, *p  = .*997, gender, *χ^2^*(1)  = 0.11, *p  = .*746, marriage status, *χ^2^*(1)  = 0.23, *p  = .*633, and education level, *χ^2^*(2)  = 0.50, *p  = .*780. Participants’ housing opportunities had been determined by the construction schedule of different companies that the village/community authorities delegated, and some residents were able to enter a lottery to obtain an apartment when some apartments became available, while the rest who did not get any apartment still lived in the temporary shelters since while waiting for the next available batch of apartments.

#### Materials and Procedure

The questionnaire was distributed and completed at participants’ apartments or shelters, and a gift was offered as reward. The materials included the 13-item BJW scale and the 10-item resilience scale as in Study 1. The two different living conditions were considered to naturally prime different temporal perspectives. Specifically, we assumed that those moving into the new apartments would be able to focus on their longer term futures, while those living in the temporary shelters would be more likely to be focused on short-term concerns, less able to envision their longer term future. We included three items about FP (as in Study 1) to check the validity of this assumption, and an independent *t*-test revealed that those in the new apartments (2.36±0.74) were higher in FP than those staying in the temporal shelters (2.11±0.87), *t*(215)  = 2.21, *p*  = .028. Meanwhile, we also asked the participants to evaluated how severely were they hurt in the disaster on a 1–4 Likert scale (1 = not at all, 4 =  severely), and found no difference between the two groups (*M* = 1.28±0.56 vs. *M* = 1.26±0.52), *t*(209)  = 0.21, *p*  = .834.

### Results

As presented on the right side of Table 1, GBJW was positively correlated with FP and resilience while PBJW was not. A paired-sample *t*-test showed that GBJW (*M* = 4.22±0.93) was stronger than PBJW (*M* = 3.92±0.95), *t*(210)  = 4.99, *p*  = .001.

Hierarchical regression analyses were used to determine whether GBJW and PBJW were related to resilience differently across different temporal conditions. To test the moderation effect of FP on BJW and resilience, FP × GBJW (or PBJW) was entered into the regression after demographic variables, while FP and GBJW (or PBJW) were controlled. Results of the first analysis concerned with GBJW showed that GBJW, *β* = 0.58, *t* = 2.38, *p*  = .019, but not FP, *β* = 0.016, *t* = 0.21, *p*  = .836, predicted resilience. The FP x GBJW interaction term was a marginally significant predictor of resilience over and above these individual terms, providing some support for our hypothesis that FP would marginally moderate the effects of GBJW, *β* = 0.12, *t* = 1.81, *p*  = .071. In contrast, the analysis concerned with PBJW showed that that neither PBJW, *β* = 0.23, *t* = 0.93, *p*  = .356, FP, *β* = 0.01, *t* = 0.15, *p*  = .880, nor the interaction term, *β* = 0.12, *t* = 0.49, *p*  = .625, predicted resilience.

**Figure 2:**
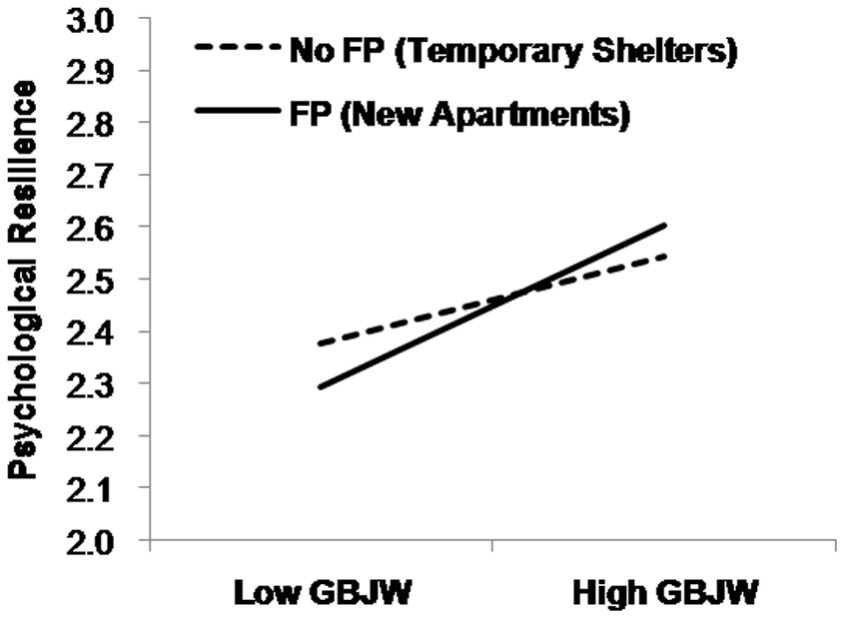
Relationship of disaster survivors’ GBJW and psychological resilience, depending on the priming of FP (Study 2). GBJW  =  general belief in a just world, FP  =  Future Perspective.

As shown in Figure 2, we examined the marginal interaction between FP and GBJW by relating GBJW to psychological resilience among survivors with FP (in new apartments) and no FP (in temporary shelters). As expected, GBJW, *β* = 0.26, *t*  = 1.99, *p*  = .050), but not PBJW, *β* = 0.01, *t*  = 0.06, *p*  = .952, predicted resilience among those with FP, while neither GBJW, β = –0.01, *t* = –0.06, *p*  = .951, nor PBJW, *β* = 0.06, *t*  = 0.51, *p*  = .612, predicted resilience among those with no FP.

### Discussion

The present results replicate and extend those of Study 1. The adult earthquake survivors in the present study, like the adolescent schoolchildren of Study 1, believed the world was more just generally than to themselves personally. As in Study 1, GBJW was positively related to resilience among participants who were strongly (vs. weakly) focused on the future. This shows that the greater strength of GBJW, and its psychological function in combination with FP, holds true whether participants are living the lives of typical schoolchildren or have been rendered homeless by a natural disaster. Further, the moderation effect of FP holds true whether it operationalized in terms of chronic individual differences within a correlational design (Study 1) or as situationally determined differences in a quasi-experimental design (Study 2). A notable difference is that in Study 2, PBJW did not predict psychological resilience among earthquake survivors, whereas it did predict resilience among the schoolchildren of Study 1. This pattern of results suggests that GBJW, relative to PBJW, is especially important to well-being for people who are confronting very adverse, unjust outcomes in their own lives – perhaps because justice currently received by others provides hope that in the future, the self, too, will receive justice. Also of note, being placed in new accommodation did not, by itself, predict psychological resilience; only when participants felt that others get what they deserve did it appear to be helpful. Perhaps, improvement in the circumstances of victims of unjust outcomes aids adjustment only when they are interpreted through the lens of just-world belief.

## General Discussion

Results from the current studies show that at as we predicted, GBJW was more strongly endorsed than PBJW. These results differ markedly from a number of studies conducted in Western populations, in which PBJW has been shown to be generally stronger than GBJW [28], [29], [30], [31]. However, they are consistent with some of our other findings in studies of Chinese participants. As well as being strongly endorsed in this population, GBJW predicted psychological resilience, over and above any effect of PBJW. In fact, PBJW was related to well-being for Chinese schoolchildren (Study 1) but not for survivors of the Wenchuan earthquake (Study 2). Studies conducted with WEIRD samples generally show that PBJW (vs. GBJW) has a stronger relationship with well being [40]. Together, the results concerning the strength and apparent function of GBJW illustrate that findings obtained with relatively privileged Westerners cannot be generalized to other populations.

The most novel finding of the present investigation is that the relationship between GBJW and resilience is moderated by temporal perspective. In both studies, the relationship between GBJW and resilience was significant among people who had adopted a FP, and marginal (Study 1) or non-significant (Study 2) among those who had not. Holding across two different research designs and operationalizations of FP, this result supports our prediction, derived from just motive theory, that just-world beliefs are especially important for psychological adjustment among people who are focused on their future [5], [6], [7], [8], 9.

Previous studies have shown that the justice motive is activated when people consider the future, with a range of consequences for motivation and social judgment. For example, a recent study shows that observers who believe that people generally get what they deserve perceive that the innocent victims of misfortune will nonetheless experience ultimate justice – that is, derive meaning and enhanced well being from their current travails [10]. Similarly, general justice beliefs have been found to be associated with enhanced self-regulation and willingness to invest resources such as time and effort in the pursuit of long-term goals [33], [34], [41], [42], [43]

The present studies are unique in showing that just-world beliefs are associated with an index of psychological well-being more strongly when people are focused on the future. Although new, this finding is fully consistent with original statements of just-world theory, in which faith in justice was seen as adaptive largely because it gives people reassurance in the future, they will receive the outcomes they deserve [8], [9].

Since the association of GBJW and resilience was contingent on FP, we cannot conclude that GBJW always has an adaptive function for the populations from which our participants were drawn. This means that characteristics of Chinese culture, such as collectivism, or holistic cognition, are not sufficient for GBJW to be adaptive. Disadvantaged living conditions may also be important, as suggested by recent findings that people who have endured difficult and unjust circumstances may be especially likely to benefit from GBJW as opposed to PBJW [16], [17], [23], [24], [25], [26], [27]. One theoretical explanation for this is that GBJW provides disadvantaged people a means of compensatory control, entailing that existing social arrangements are fair and deserved and that good outcomes may be obtained through effort and merit [42], [43]. GBJW depicts an orderly and controllable world, which helps preserve a sense of control even when personal control vanishes [32], [44], [45], [46]. An abundant literature suggests that such beliefs can serve a palliative function for people who lack the experience of justice in their lives [34], [41], [42], [43]. In this case, it is perhaps not surprising that GBJW is especially useful when such people focus on the future, facilitating the hope that the present injustices in their own lives may be replaced by the justice that people are believed to experience, generally.

Culture, nonetheless, may be important. In the case of collectivistic cultures where people have to confront widespread disasters [47] and socioeconomic obstruction [48], a robust GBJW could help such individuals as the Confucians in harsh realities pursue a good life through their own ability, power, and effort, or help such individuals as Taoists spiritually enjoy the meaning of transcending adversity [49], [50]. Of course, we notice that GBJW is not specifically restricted to collectivistic cultures, and a robust GBJW has also been shown by cross-situational and cross-cultural investigations to be present in a large amount of Western literature [7], [14], [28], [51], [52], [53], [54], [55]. Further research is required to disentangle the potential moderating roles of culture and disadvantage. For example, individual differences in cultural variables, such as collectivism, philosophy and cognition, can be examined as potential moderators along with differences in (dis)advantaged personal circumstances.

Whatever the ultimate explanation for the possible benefits of GBJW for people with a future perspective, it is important to note that they are likely to have a downside. Specifically, it can lead to negative responses to victims [5], [6]. Thus, characteristics that seem intuitively related to rational thought (long-term planning) and to goodness (idealism about justice) might lead to both resilient responses that benefit the self, and defensive responses that revictimize unfortunate others. In other words, self resilience and social maladjustment could coexist as two sides of the same coin [56], so that there is a social cost of the personal benefits of GBJW. Future research could test the possibility that, under specific conditions, anticipated GBJW leads to increased self-resilience and decreased empathy for victims at the same time.

Taken together, a major strength of the two studies presented here is that converging results were found with different samples and methodological approaches. The studies nonetheless have important limitations. Not least of which, neither study is experimental. Study 1 relies on a cross-sectional examination of relationships between individual differences in GBJW, FP, and resilience; Study 2 relies on a quasi-experimental design in which situational variance in FP is examined as a moderator of the relationship between GBJW and resilience. Further, both studies rely on a self-report measure of resilience. Future studies should attempt experimental investigations of FP, and other measures of resilience, ideally including a more objective or behavioral component.

Despite these limitations, the studies provide initial evidence that GBJW is more important in important sections of the global community than is apparent from two decades of research conducted largely with relatively privileged samples of Westerners. In the present studies it appears to be endorsed more strongly than PBJW, and more strongly associated with resilience among people whose temporal perspectives are oriented toward the future. Although future studies are required to examine whether the relationship between GBJW and adjustment observed in the present studies is causal, the present results suggest that perhaps, GBJW is especially useful to people whose cultural background, and/or their personal circumstances, create a lived experience quite different from the phenomenological “world of the self’ that is characteristic of the Westerners who were focal in the original statements of just motive theory [8], [9].

## References

[1] Gilbert DT, Wilson TD (2007) Prospection: Experiencing the future. Science 317: 1351–1354. 17823345 10.1126/science.1144161

[2] Atance CM, O'Neill DK (2001) Episodic future thinking. Trends in Cognitive Sciences 5: 533–539. 11728911 10.1016/s1364-6613(00)01804-0

[3] Trope Y, Liberman N (2003) Temporal construal. Psychological Review 110: 403–421. 12885109 10.1037/0033-295x.110.3.403

[4] Gilbert DT, Brown RP, Pinel EC, Wilson TD (2000) The illusion of external agency. Journal of Personality and Social Psychology 79: 690–700. 11079235 10.1037//0022-3514.79.5.690

[5] Hafer CL (2000) Investment in long-term goals and commitment to just means drive the need to believe in a just world. Personality and Social Psychology Bulletin 26: 1059–1073.

[6] Hafer CL, Bègue L, Choma BL, Dempsey JL (2005) Belief in a just world and commitment to long-term deserved outcomes. Social Justice Research 18: 429–444.

[7] Sutton RM, Winnard EJ (2007) Looking ahead through lenses of justice: The relevance of just-world beliefs to intentions and confidence in the future. British journal of social psychology 46: 649–666. 17877857 10.1348/014466606X166220

[8] Lerner MJ, Miller DT (1978) Just world research and the attribution process: Looking back and ahead. Psychological Bulletin 85: 1030–1051.

[9] Lerner MJ (1980) The belief in a just world: A fundamental delusion. New York and London: Plenum Press. 11 p.

[10] Anderson J, Kay A, Fitzsimons G (2010) In search of the silver lining: The justice motive fosters perceptions of benefits in the later lives of tragedy victims. Psychological Science 21: 1599–1604. 20959512 10.1177/0956797610386620

[11] Bal M, van den Bos K (2012) Blaming for a Better Future: Future Orientation and Associated Intolerance of Personal Uncertainty Lead to Harsher Reactions Toward Innocent Victims. Personality and Social Psychology Bulletin 38: 835–844. 22492551 10.1177/0146167212442970

[12] Kay AC, Jimenez MC, Jost JT (2002) Sour grapes, sweet lemons, and the anticipatory rationalization of the status quo. Personality and Social Psychology Bulletin 28: 1300–1312.

[13] Gaucher D, Hafer CL, Kay AC, Davidenko N (2010) Compensatory rationalizations and the resolution of everyday undeserved outcomes. Personality and Social Psychology Bulletin 36: 109–118. 19915098 10.1177/0146167209351701

[14] Furnham A (2003) Belief in a just world: research progress over the past decade. Personality and Individual Differences 34: 795–817.

[15] Hafer C, Olson J (1998) Individual differences in beliefs in a just world and responses to personal misfortune. In: Montada L, Lerner M, editors. Responses to victimizations and belief in the just world. New York: Plenum. pp. 65–86.

[16] Hafer CL, Correy BL (1999) Mediators of the Relation Between Beliefs in a Just World and Emotional Responses to Negative Outcomes. Social Justice Research 12: 189–204.

[17] Tomaka J, Blascovich J (1994) Effects of justice beliefs on cognitive appraisal of and subjective, physiological, and behavioral responses to potential stress. Journal of Personality and Social Psychology 67: 732–732. 7965617 10.1037//0022-3514.67.4.732

[18] Henrich J, Heine SJ, Norenzayan A (2010) Most people are not WEIRD. Nature 466: 29–29. 20595995 10.1038/466029a

[19] Dalbert C (1998) Belief in a just world, well-being, and coping with an unjust fate. In: Montada L, Lerner MJ, editors. Responses to Victimization and Belief in a Just World. New York: Plenum. pp. 87–105.

[20] Cubela Adoric V (2004) Belief in a just world and young adults’ ways of coping with unemployment and the job search. In: Dalbert C, Sallay H, editors. The Justice Motive in Adolescence and Young Adulthood: Origins and Consequences. London: Routledge. pp. 189–214.

[21] Dzuka J, Dalbert C (2006) The belief in a just world and subjective well-being in old age. Aging & Mental Health 10: 439–444. 16938679 10.1080/13607860600637778

[22] Dzuka J, Dalbert C (2007) Student violence against teachers: Teachers' well-being and the belief in a just world. European Psychologist 12: 253–260.

[23] McParland J, Knussen C (2010) Just world beliefs moderate the relationship of pain intensity and disability with psychological distress in chronic pain support group members. European Journal of Pain 14: 71–76. 19121590 10.1016/j.ejpain.2008.11.016

[24] Wu S-T, Wang L, Zhou M-J, Wang W-Z, Zhang J-X (2009) Belief in a Just World and Subjective Well-Being: Comparing Disaster Sites with Normal Areas. Advances in Psychological Science 17: 579–587.

[25] Zhu ZH, Wu ST, Li J, Shi ZB, Wang WZ (2010) Belief in a Just World and Satisfaction of Teachers in 5.12 Sichuan Earthquake. Chinese Journal of Clinical Psychology 18: 79–84.

[26] Wu MS, Yan X, ZHou C, Chen Y, Li J, et al (2011) General belief in a just world and resilience: Evidence from a collectivistic culture. European Journal of Personality 25: 431–442.

[27] Wu MS, Kamble S, Khachatryan N, Wang Y, Jiang Y, et al.. (2012) Robust Effect of Justice for Others among the Disadvantaged: Evidence from Explicit and Implicit Perspectives. The 14th Biennial Conference of International Society for Justice Research (ISJR). Reshion Lezion, Israel.

[28] Sutton RM, Douglas KM (2005) Justice for all, or just for me? More evidence of the importance of the self-other distinction in just-world beliefs. Personality and Individual Differences 39: 637–645.

[29] Lipkus IM, Dalbert C, Siegler IC (1996) The importance of distinguishing the belief in a just world for self versus for others: Implications for psychological well-being. Personality & Social Psychology Bulletin 22: 666–677.

[30] Dalbert C (1999) The world is more just for me than generally: About the personal belief in a just world scale's validity. Social Justice Research 12: 79–98.

[31] Bègue L, Bastounis M (2003) Two spheres of belief in justice: Extensive support for the bidimensional model of belief in a just world. Journal of Personality 71: 435–463. 12762422 10.1111/1467-6494.7103007

[32] Kay AC, Whitson JA, Gaucher D, Galinsky AD (2009) Compensatory control: Achieving order through the mind, our institutions, and the heavens. Current Directions In Psychological Science 18: 264–268.

[33] Laurin K, Fitzsimons GM, Kay AC (2011) Social disadvantage and the self-regulatory function of justice beliefs. Journal of Personality and Social Psychology 100: 149–171. 21058869 10.1037/a0021343

[34] Jost JT, Pelham BW, Sheldon O, Sullivan BN (2003) Social inequality and the reduction of ideological dissonance on behalf of the system: Evidence of enhanced system justification among the disadvantaged. European Journal of Social Psychology 33: 13–36.

[35] Otto K, Dalbert C (2005) Belief in a just world and its functions for young prisoners. Journal of Research in Personality 39: 559–573.

[36] Campbell-Sills L, Stein M (2007) Psychometric analysis and refinement of the Connor-davidson Resilience Scale (CD-RISC): Validation of a 10-item measure of resilience. Journal of Traumatic Stress 20: 1019–1028. 18157881 10.1002/jts.20271

[37] Connor K, Davidson J (2003) Development of a new resilience scale: the Connor-Davidson Resilience Scale (CD-RISC). Depression and Anxiety 18: 76–82. 12964174 10.1002/da.10113

[38] Yu S-H, Lin Y-C, Huang C-L, Hwang K-K, Chang J-H (2010) The Relationship between Long-Term Orientation and Psychological Adjustment. Formosa Journal of Mental Health 23: 347–375.

[39] Aiken LS, West SG (1991) Multiple regression: Testing and interpreting interactions. Thousand Oaks, CA: Sage.

[40] Nudelman G (2013) The Belief in a Just World and Personality: A Meta-analysis. Social Justice Research 26: 105–119.

[41] Jost J, Hunyady O (2002) The psychology of system justification and the palliative function of ideology. European review of social psychology 13: 111–153.

[42] Jost JT, Banaji MR, Nosek BA (2004) A decade of system justification theory: Accumulated evidence of conscious and unconscious bolstering of the status quo. Political Psychology 25: 881–919.

[43] Jost JT, Hunyady O (2005) Antecedents and consequences of system-justifying ideologies. Current Directions in Psychological Science 14: 260–265.

[44] Kay AC, Jost JT (2003) Complementary justice: Effects of" poor but happy" and" poor but honest" stereotype exemplars on system justification and implicit activation of the justice motive. Journal of personality and social psychology 85: 823–837. 14599247 10.1037/0022-3514.85.5.823

[45] Kay AC, Jost JT, Mandisodza AN, Sherman SJ, Petrocelli JV, et al (2007) Panglossian ideology in the service of system justification: How complementary stereotypes help us to rationalize inequality. Advances in experimental social psychology 39: 305–358.

[46] Kay AC, Gaucher D, Napier JL, Callan MJ, Laurin K (2008) God and the government: Testing a compensatory control mechanism for the support of external systems. Journal of personality and social psychology 95: 18–35. 18605849 10.1037/0022-3514.95.1.18

[47] Fincher CL, Thornhill R, Murray DR, Schaller M (2008) Pathogen prevalence predicts human cross-cultural variability in individualism/collectivism. Proceedings of the Royal Society B: Biological Sciences 275: 1279–1285. 18302996 10.1098/rspb.2008.0094 PMC2602680

[48] Inglehart R, Welzel C (2005) Modernization, Cultural Change, and Democracy. New York: Cambridge University Press. 64 p.

[49] Fung Y-L (1985) Short history of Chinese philosophy. New York: The Free Press.

[50] Zhang G, Veenhoven R (2008) Ancient Chinese philosophical advice: Can it help us find happiness today? Journal of Happiness Studies 9: 1–19.

[51] Bond M, Leung K, Au A, Tong K, Chemonges-Nielson Z (2004) Combining social axioms with values in predicting social behaviours. European Journal of Personality 18: 177–191.

[52] Bond M, Leung K, Au A, Tong K, de Carrasquel S, et al (2004) Culture-Level Dimensions of Social Axioms and Their Correlates across 41 Cultures. Journal of Cross-Cultural Psychology 35: 548–570.

[53] Lucas T, Zhdanova L (2010) Justice Beliefs for Self and Others: Links to Well-Beijing in African Americans. International Society for Social Justice Research 13th Biennial Conference. Banff, Alberta, Canada.

[54] Furnham A (1993) Just world beliefs in twelve societies. The Journal of Social Psychology 133: 317–329.

[55] Sutton RM, Douglas KM, Wilkin K, Elder TJ, Cole JM, et al (2008) Justice for whom, exactly? Beliefs in justice for the self and various others. Personality and Social Psychology Bulletin 34: 528–541. 18340035 10.1177/0146167207312526

[56] Bonanno GA, Rennicke C, Dekel S (2005) Self-enhancement among high-exposure survivors of the September 11th terrorist attack: Resilience or social maladjustment? Journal of Personality and Social Psychology 88: 984–998. 15982117 10.1037/0022-3514.88.6.984

